# Different culture media modulate growth, heterogeneity, and senescence in human mammary epithelial cell cultures

**DOI:** 10.1371/journal.pone.0204645

**Published:** 2018-10-01

**Authors:** Jonathan K. Lee, Jessica Bloom, Arantzazu Zubeldia-Plazaola, James C. Garbe, Martha R. Stampfer, Mark A. LaBarge

**Affiliations:** 1 Biological Systems and Engineering Division, Lawrence Berkeley National Laboratory, Berkeley, CA, United States of America; 2 Department of Population Sciences, Beckman Research Institute, City of Hope, Duarte, CA, United States of America; 3 Institut d’Investigacions Biomediques August Pi i Sunyer, University of Barcelona, Barcelona, Spain; The Ohio State University, UNITED STATES

## Abstract

The ability to culture normal human mammary epithelial cells (HMEC) greatly facilitates experiments that seek to understand both normal mammary cell biology and the many differences between normal and abnormal human mammary epithelia. To maximize *in vivo* relevance, the primary cell culture conditions should maintain cells in states that resemble *in vivo* as much as possible. Towards this goal, we compared the properties of HMEC strains from two different reduction mammoplasty tissues that were grown in parallel using different media and culture conditions. Epithelial organoids were initiated into three different media: two commonly used serum-free-media, MCDB 170-type (e.g. MEGM) and WIT-P, and a low stress media, M87A. Growth, lineage heterogeneity, p16 protein expression, and population doublings to senescence were measured for each culture condition. MCDB 170 caused rapid senescence and loss of heterogeneity within 2 to 3 passages, but some cultures went through the 1 to 2 month process of selection to generate clonal finite post-selection post-stasis cells. WIT-P caused impressive expansion of luminal cells in 2^nd^ passage followed by their near complete disappearance by passage 4 and senescence shortly thereafter. M87A supported as much as twice the number of population doublings compared to either serum-free medium, and luminal and myoepithelial cells were present for as many as 8 passages. Thus, of the three media compared, WIT-P and MCDB 170 imposed rapid senescence and loss of lineage heterogeneity, phenotypes consistent with cells maintained in high-stress conditions, while M87A supported cultures that maintained multiple lineages and robust growth for up to 60 population doublings. In conjunction with previous studies examining the molecular properties of cultures grown in these media, we conclude that M87A medium is most able to support long-term culture of multiple lineages similar to *in vivo* conditions, thereby facilitating investigations of normal HMEC biology relevant to the mammary gland *in situ*.

## Introduction

Experimental examination of normal human mammary epithelial cell (HMEC) behavior, and how normal cells acquire abnormal properties, can be facilitated by *in vitro* culture systems that accurately model *in vivo* biology. The breast consists of a complex admixture of many distinct cell types, e.g., epithelial, adipose, mesenchymal, endothelial. The epithelial cells are responsible for the differentiated mammary function of lactation and are also the origin of the vast majority of human breast cancers. The mammary epithelium consists of at least two, broadly classified types; the luminal epithelial and myoepithelial cell lineages. Cultured HMEC have been employed in a wide variety of studies examining the normal processes governing growth, differentiation, self-organization, aging, and senescence, and how these normal processes are altered during immortal and malignant transformation[1–20]. The effects of HMEC growth in the presence of extracellular matrix material, other cell types, and 3D culture, has been compared with growth on plastic [10]. Cultured HMEC, starting with normal cells, can provide an experimentally tractable system to examine factors that may propel or prevent human aging and carcinogenesis. The growth media and methodology used to initiate and maintain primary HMEC strains are crucial factors that directly impact the properties of the cultured cells and the interpretation of experiments.

Arguably the cell culture media most commonly used to support HMEC growth *in vitro* is serum-free MCDB 170-type media developed in the 1980s by Ham and Stampfer [7], which has been commercially available since 1986 (e.g. Lonza MEGM). Normal HMEC strains established in MCDB 170-type media have typically undergone a selection process within 2–4 passages, whereby a majority of the HMEC cease net growth at stress-associated senescence (stasis), coupled with elevated levels of the cyclin-dependent kinase inhibitor p16^INK4a^ [1, 7, 16, 18]. However, in MCDB 170-type media, rare cells may overcome stasis by epigenetic silencing of p16, along with many other epigenetic and transcriptional changes [1, 12, 21]. These p16(-) post-selection post-stasis HMEC, later also referred to as v(ariant) HMEC [22], are uniformly basal, have metaplastic properties, and have been suggested to be on a pathway to metaplastic tumors [1, 3, 12, 21, 23–25]. Thus, although they are sold commercially as ‘normal HMEC’, they exhibit many significant molecular differences from normal pre-stasis HMEC. Serum-free WIT media was developed with the intent of enabling culture of normal and isogenic transformed HMEC. It also was reported to better support the maintenance of luminal cells when combined with a proprietary form of tissue culture plastic (TCP), compared to MCDB 170-type media on standard TCP [8], thereby partly addressing the issue of maintaining the luminal phenotype beyond initial passages. M87A-type media was developed to provide a lower-stress culture environment, i.e., after the rapid induction of p16 in MCDB 170-type media was recognized [3], to allow increased proliferation by postponing induction of p16. M87A was reported to support pre-stasis HMEC growth for as much as 60 population doublings (PD), and to maintain luminal cells for as many as 8 passages (~30 PD)(5). While all these media are capable of supporting growth of multiple lineages for at least the first or second passage, a more rigorous comparison of these media beyond the earliest passages has been lacking.

To compare the impact of culture media on normal pre-stasis HMEC growth and lineage representation, we examined the growth and phenotypes of HMEC cultures derived from two different individuals, age 19 and 45, grown in the three different culture media (MCDB 170, WIT-P on Primaria^TM^ TCP, and M87A) from passage 2 until they entered stasis. M87A supported 2–3 fold more PD of pre-stasis HMEC compared to MCDB 170 or WIT-P, which both led to rapid onset of stasis. Flow cytometry and immunofluorescence analyses of mammary epithelial lineage markers revealed differences in the abilities of the three-different media to maintain multiple lineages in pre-stasis culture, with HMEC grown in M87A being the only cultures that contained multiple lineages beyond the first few passages. The ability to maintain lineage diversity is crucial for enabling use of cultured HMEC as models of normal *in vivo* biology and of the process of malignant transformation.

## Material and methods

### Cell culture

**Fig 1:**
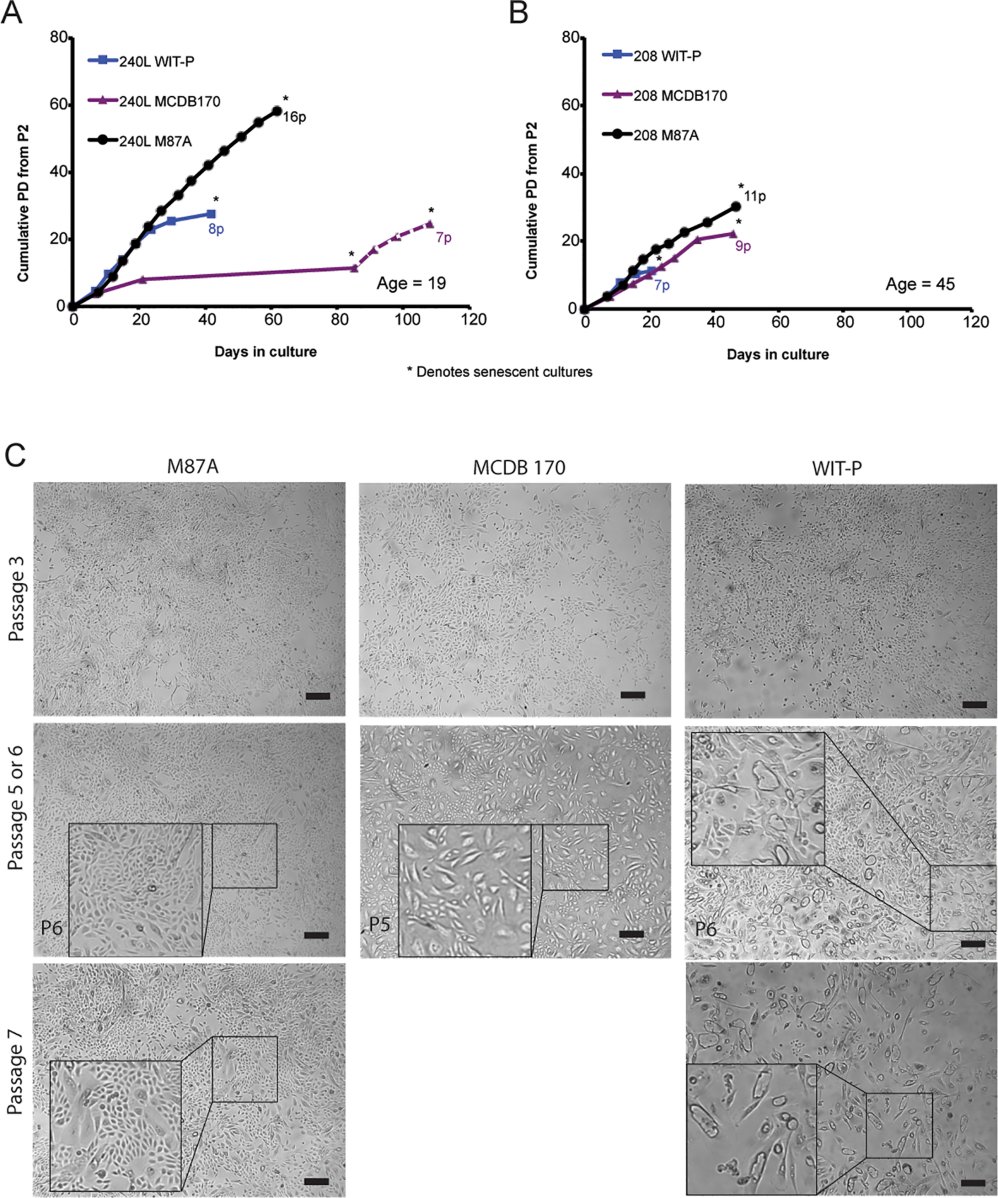
Comparison of HMEC growth in pre-stasis strains established in parallel from two different breast tissue samples using three different media. Curves show PD as a function of time for HMEC strains generated from specimens (A) 240L and (B) 208, aged 19 and 45 years respectively. Blue squares denote cultures grown in WIT-P, purple triangles denote growth in MCDB 170, and black circles denote growth in M87A. In (A), MCDB 170 led to rapid induction of stasis by 4p, followed by emergence of a clonal post-selection post-stasis culture denoted by the dashed line. Asterisks denote when cultures ceased net growth. (C) Phase images show pre-stasis 240L cells grown in M87A, MCDB 170, or WIT-P media at passages ranging from 3-7p. Bars represent 100**μ**m. Note the prominent appearance of large vacuolated cells by 5p and 6p in MCDB 170 and WIT-P, respectively. 5p and 7p in MCDB 170 and WIT-P were the last images shown because both cultures completely ceased growth by the following passage.

HMEC from specimens 240L (batch B) and 208 were obtained from reduction mammoplasty tissue of women aged 19 and 45 years respectively. Deidentified tissue specimens were obtained in accordance with IRB and human use policies of the Lawrence Berkeley National Lab. Both HMEC were initiated in primary culture from organoids and grown until stasis and/or replicative senescence in serum-free WIT-P (Stemgent), serum-free MCDB 170 with supplements (MEBM, Lonza, Walkersville, MD), or serum containing M87A with supplements, as described in supporting information file S1 File and in references [3, 7, 8, 9, 16, 18, 19, 26]. Total PD were calculated beginning at passage 2 using the equation $PD=\log_{2}\left(\frac{N_{final}}{N_{initial}}\right)$. N is the number of cells counted. A known number of cells were seeded at the start of every passage in each dish, and cells were counted at the point the dish became sub-confluent. We define a sub-confluent culture as a proliferative culture that has not yet covered the entire dish; Fig 1C left column shows examples of 80–90% sub-confluent cultures in of 240L cells in M87A media at p6 and p7. Each viable cell count was done in triplicate using a hemocytometer.

### Immunofluorescence

HMEC cultures were grown on glass coverslips and fixed at passages 2, 4, and 7 in 1:1 methanol/acetone at -20°C for 15 minutes. Fixed HMEC were blocked in 1X PBS, 5% normal goat serum, and 0.1% Triton X-100 (Invitrogen) overnight, then stained with polyclonal rabbit anti-Keratin 14 (1:1000, Covance PRB-155P), and mouse anti-Keratin 19 (1:100, Invitrogen) in blocking buffer and incubated at 4C overnight. Following 3 washes in 1X PBS for 10 min, HMEC were incubated with fluorescent secondary antibodies for 2 hours at room temperature with 1:200 Alexa Fluor 488 Goat anti-Mouse IgG2a(Invitrogen A#21131) and 1:200 Alexa Fluor 568 Goat anti-Rabbit IgG(Invitrogen #A11011) and DAPI. Cells were imaged using a Zeiss LSM710 confocal microscope with Zen acquisition software. Marker-based watershed segmentation was performed as previously described [13] using MatLab software from Mathworks inc. To quantify heterogeneity with respect to lineage, Shannon Diversity Indexes, were calculated for each culture as a function of passage according to the equation H = $-\sum_{s=1}^{s}p_{i}lnp_{i}$

### Immunohistochemistry

Cells were washed twice with PBS and fixed for 30 min with 4% paraformaldehyde, then permeablized with 0.1% Triton X-100 for 5 min, blocked for 30 min in PBS containing 5% normal goat serum, and incubated with p16 antibody (Santa Cruz Biotech, SC-56330, clone JC8) for 60 min. Antibody binding was visualized using peroxidase mouse ABC kit and DAB substrate kit (Vector Labs, Burlingame, CA).

### Flow cytometry

Subconfluent cells were trypsinized and fixed in 2% paraformaldehyde. Cells were blocked in FACS buffer (2.5% BSA, 1mM EDTA in 1XPBS) and stained with CD227-FITC (BD Biosciences, clone HMPV, 1:50) and CD10-PE (BioLegend, clone HI10a, 1:100) in FACS buffer for 25 min on ice protected from light, washed in PBS, and analyzed using FACS Calibur (Becton Dickinson). FlowJo X was used to for computer analysis.

### Statistical analysis

**Fig 2:**
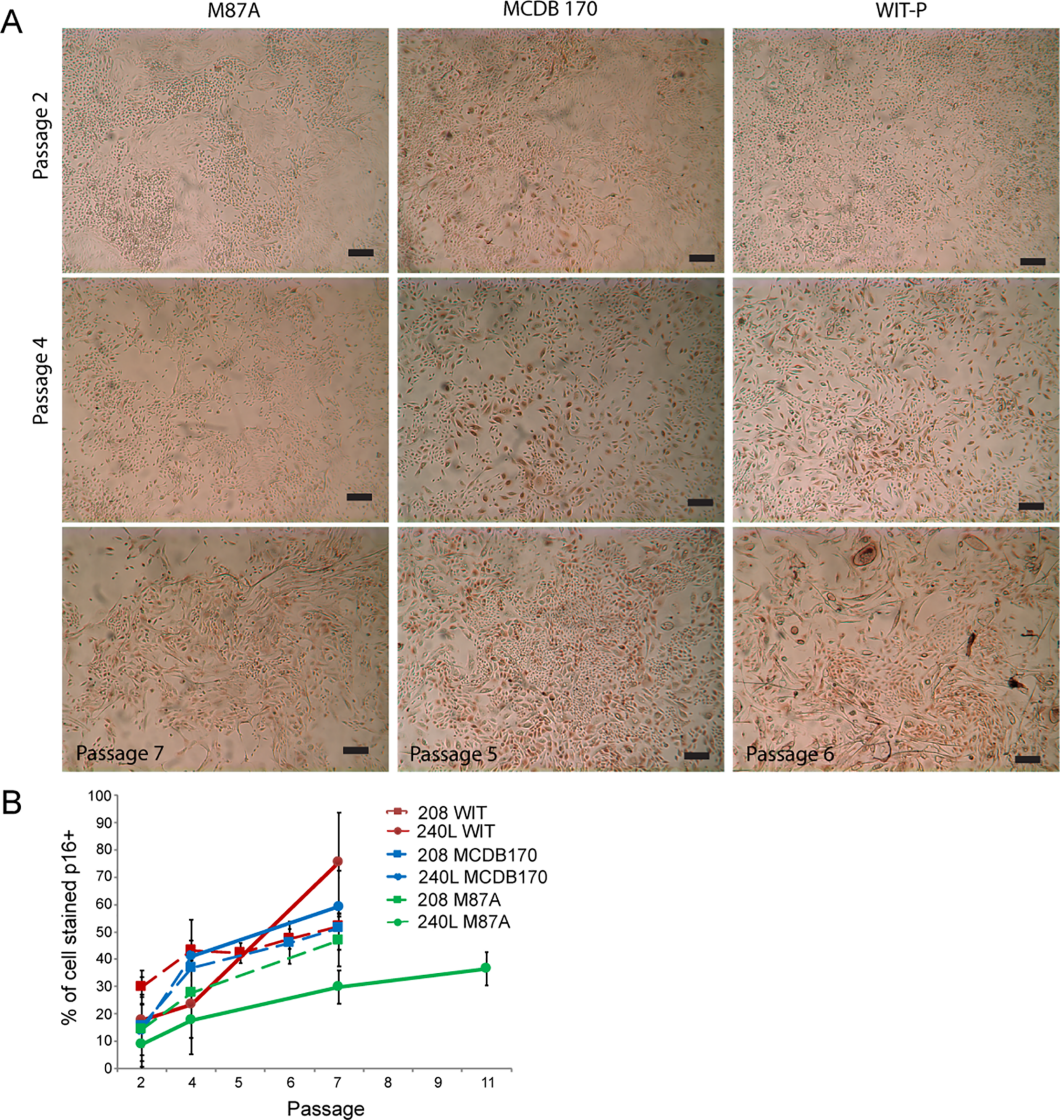
Immunohistochemical examination of p16 protein expression in pre-stasis HMEC 240L in three different growth media. (A) The presence of p16 protein is detectable by brown-colored precipitate formed by horse radish peroxidase reactions. Images are shown of HMEC 240L grown in M87A, MCDB 170, and WIT-P at 2p and 4p. Cells in MCDB 170 and WIT-P are shown also at p6 and p5, respectively, because both of those media cause significant number of p16+ cells as early as those passages, whereas M87A is shown at 7p. Bars represent 100**μ**m. (B) Line graphs showing quantification of percent p16 stained cells/total as a function of passage for strain 240L (solid line) and 208 (dashed line) cultured in M87A (green), MCDB 170 (blue), or WIT-P (red). From 3–12 images of each specimen at the indicated passages in all three media were counted. Areas under the curve (AUC) are reported for each media (240L,208): M87A (97.0, 153.7), MCDB 170 (205.4, 183.7), WIT-P (189.1, 209.9).

Graphpad Prism 5.0 for PC and Matlab were used for all statistical analysis. To compare two population distributions, chi-square and t-tests were performed. Means were determined with technical triplicates of biological replicates. Significance was established when *p < 0.05 and **p < 0.01. For p16 staining curves, 163 images of cultures stained for p16 protein were counted in a single-blinded fashion using Image J counting plugin, by two different individuals not directly associated with this project. Results of both individuals were compared, were found to agree, and we present one dataset in Fig 2B. Area under the curve was calculated with Graphpad Prism 5.0.

## Results

### Growth rates and senescence

To determine the impact of cell culture media on growth of pre-stasis HMEC, epithelial organoids isolated from reduction mammoplasty tissues from two women were used to generate pre-stasis strains in three different culture media: MCDB 170, WIT-P, and M87A. Growth curves were generated starting with (p)assage 2 cells from both individuals, specimens 240L batch Y (Fig 1A) and 208 (Fig 1B). Cells in MCDB 170 and M87A media were grown on standard TCP dishes, whereas cells in WIT-P were grown on Primaria^TM^ dishes. Most 240L HMEC in MCDB 170 entered p16(+) stasis as early as 3p, but clonal outgrowth of p16(-) post-stasis post-selection cells was observed after about 60 days, as previously reported for HMEC grown in MCDB 170 [7]. The post-selection cells ceased net growth by 7p. Specimen 208 HMEC in MCDB 170 ceased all growth by 9p and emergence of post-selection cells was not detected even after an additional 60 days of observation. HMEC in WIT-P initially grew well, but numerous large and vacuolated cells appeared as early as 3p, with cessation of proliferation in both individuals by 7-8p. In M87A, 240L ceased growth at 16p while 208 stopped at 11p.

Morphological differences within the same strains grown in different media appeared as early as 3p. For example, cells in MCDB 170 and WIT-P showed increased numbers of large and vacuolated cells, a phenotype linked to senescence, as early as 3p, and obviously by 5-7p, compared to cultures grown in M87A (Fig 1C). Immunohistochemical staining detected expression of p16 protein in all three media by 4p, with staining most apparent in the large, senescent-appearing cells compared to the smaller, still proliferative cells (Fig 2A). In MCDB 170 media, p16-staining was apparent in cells as early as 2p, prominent by 4p and most cells were large and stained by 6p (Fig 2). In WIT-P media, cells were mostly negative for p16 at 2p, but mostly positive by 4p (Fig 2). HMEC in M87A had few p16-stained cells at 2p, still few at 4p, and only heterogeneous staining at 7p (Fig 2A). Quantification of p16 stained HMEC as a function of passage verified our observations, and the areas under the curve of HMEC grown in M87A were lower compared to MCDB 170 or WIT-P (Fig 2B). Comparison of PD potential, passage level of appearance of senescent morphologies, and p16 expression reveals that M87A supports superior growth of pre-stasis HMEC vs WIT-P and MCDB 170, which produce earlier onset of stasis.

### Maintenance of epithelial lineage diversity

**Fig 3:**
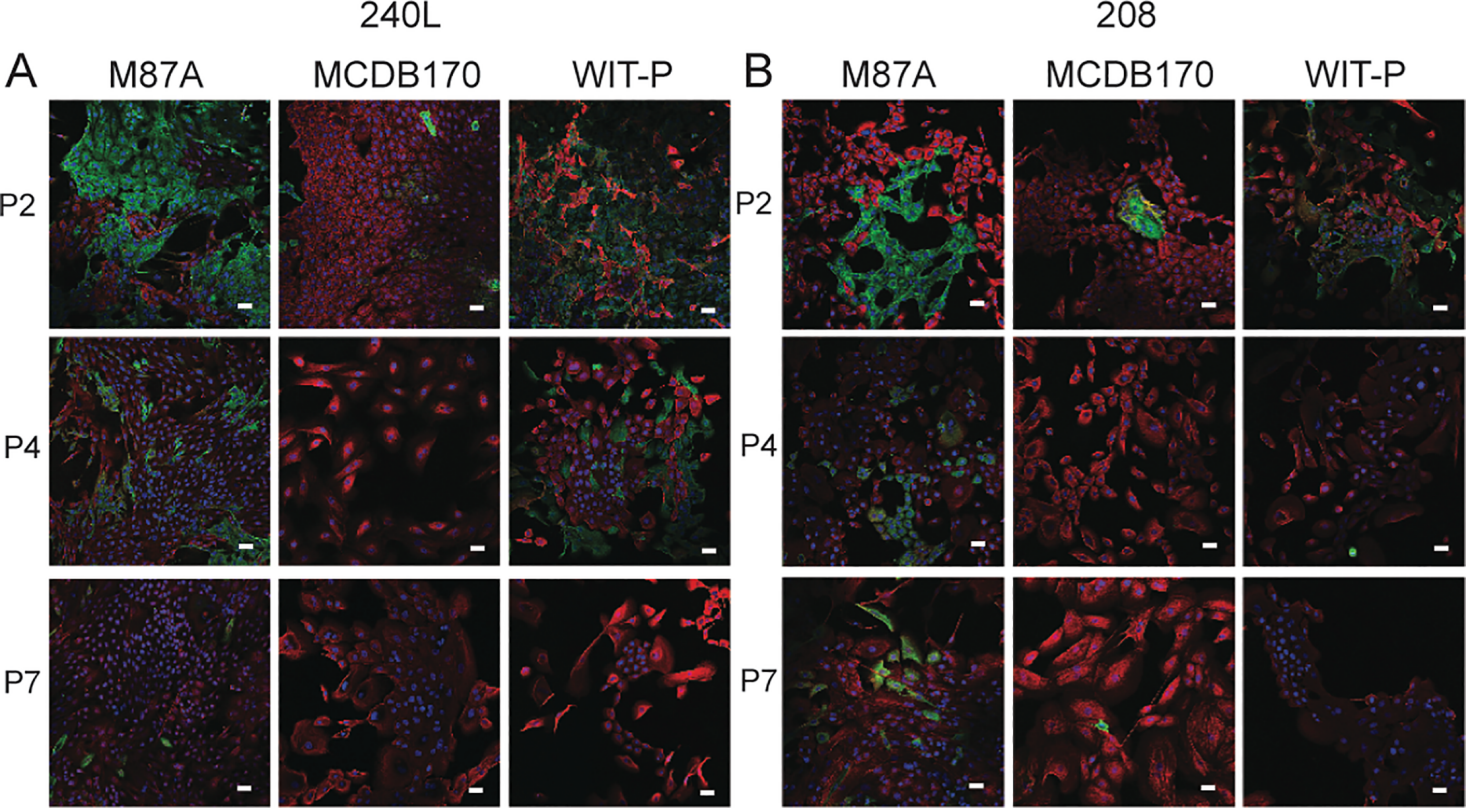
Immunofluorescence assessment of epithelial lineages in pre-stasis HMEC cultures. Cultures generated from specimens (A) 240L and (B) 208 were stained to detect expression of (K)eratin 14 (red), K19 (green), and DAPI (blue). Representative images are shown from passage 2, 4, and 7 HMEC grown in M87A, MCDB 170, and WIT-P media. Magnification bars represent 100**μ**m.

**Fig 4:**
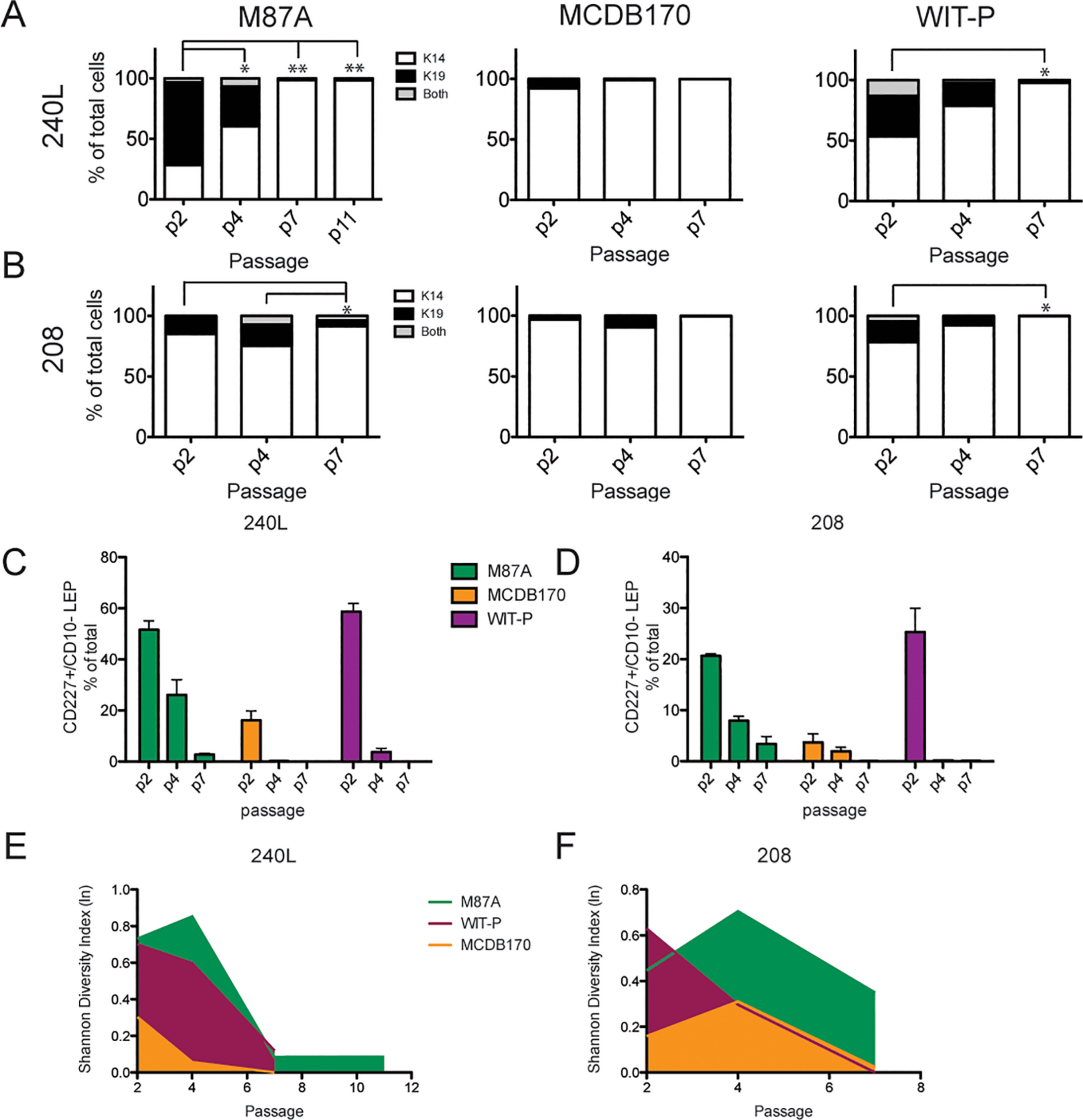
M87A maintains lineage heterogeneity in pre-stasis HMEC strains. (A and B) Bar graphs showing the proportions of K14+ MEP, K19+ LEP, or K14+/K19+ cells in cultures of specimen (A) 240L, and (B) 208, as determined with automated marker-based watershed cell segmentation from 2 replicates and 10 images from each culture condition. (C and D) Bar graphs showing the proportion of CD227+ LEP as a percentage of total cells in cultures from specimen (C) 240L or (D) 208. Error bars represent SEM. Data shown was derived from flow cytometry analysis of CD227 and CD10 expression in the pre-stasis cultures over passage, in M87A (green), WIT-P (purple), and MCDB 170 (yellow). (E and F) Integrated line plots of the Shannon Diversity Index as a function of passage for specimens (E) 240L and (F) 208 cultured in M87A (green), WIT-P (purple), and MCDB 170 (yellow). Values closest to 1.0 denote the most diversity. * and ** represent P<0.05 and P<0.01, respectively.

Maintaining the observed *in vivo* epithelial lineages in culture is crucial to understanding how the epithelium normally functions, and which cell types are impacted by pathological changes. Cultures were examined by immunofluorescence for keratin (K)14 and K19 expression, and by FACS for CD227 (Sialomucin1) and CD10 (CALLA) to assess lineage heterogeneity as a function of passage. K14 and CD10 are conventionally used as biomarkers of myoepithelial cells, while K19 and CD227 are employed as biomarkers of luminal cells. Immunofluorescence staining showed that both luminal and myoepithelial cells were present at 2p in all conditions, but with increasing passage luminal cells rapidly disappeared in MCDB 170 and to a slower extent in WIT-P cultures (Fig 3). Marker-based watershed segmentation was used to quantify K14 and K19 fluorescence in single cells. Cultures of both 240L and 208 in M87A contained cells from both lineages until at least 7p, with the luminal cells decreasing proportionately with successive passages (Fig 4A and 4B). HMEC grown in WIT-P had a large proportion of luminal cells at 2p, some large and flat K19-expressing cells at 4p, and no luminal cells by 7p. Cultures maintained in MCDB 170 showed a loss of nearly all luminal cells by 2p (Fig 4A and 4B). Flow cytometry analyses of CD227 and CD10 expression in the six cultures revealed the same pattern: CD227+ luminal cells were maintained at a higher proportion and for more passages in M87A compared to MCDB 170 or WIT-P (Fig 4C and 4D). Using the single cell data from image analysis a diversity index was calculated as a function of passage for each cell strain in all three media. Each Shannon Diversity Index value (H) represents the summation of the respective proportions of the entire population each cell type represents; thus, the larger the index value, the more diverse is the population. Comparison of the resulting H-values demonstrates that M87A maintained heterogeneity longer compared to WIT-P and MCDB 170 media (Fig 4E and 4F).

### Fetal bovine serum-free versus serum-containing M87A formulation

**Fig 5:**
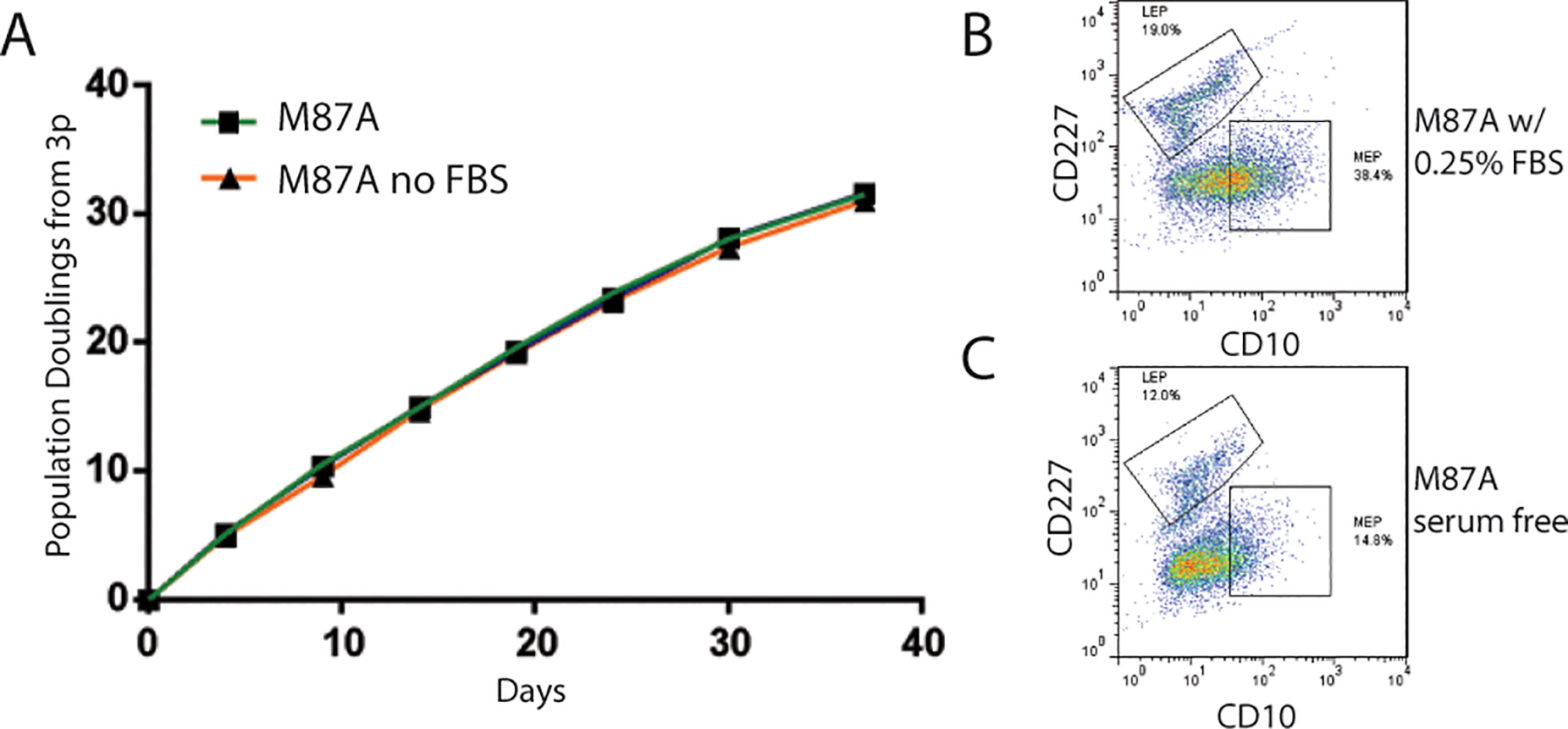
Effect of growth and lineage diversity in pre-stasis HMEC in M87A ± fetal bovine serum. (A) Curves showing PD as a function of time in days for 240L grown in M87A with and without 0.25% FBS from 3p. Growth rates were only assessed to 10p, thus the curves to not reflect cultures reaching stasis. Pseudocolor dot plots representing flow cytometry measurements of CD227 and CD10 expression in 4p HMEC cultured in (B) regular M87A media or (C) M87A without fetal bovine serum (which is normally at 0.25%). Whereas growth rates are identical with or without serum, LEP cells are lost more rapidly in serum-free M87A.

One perceived advantage of WIT-P and MCDB 170 is that they are serum-free media, although they also contain the undefined elements of bovine pituitary extract in MCDB 170 and bovine serum albumin in WIT-P. Fetal bovine serum (FBS)-free media offer some advantages over serum-containing media because there are fewer undefined elements and the composition can be less variable between batches. We therefore examined the requirement for the low level of serum (0.25%) in the M87A formulation. Growth of 240L measured from 3p showed no difference in M87A ±serum (Fig 5). However, FACS analysis of CD227 and CD10 expression revealed that HMEC populations grown in serum-free M87A contained nearly 30% fewer CD227+/CD10- luminal cells, and over 50% fewer CD227-/CD10+ myoepithelial cells at 4p compared to serum-containing M87A. These results suggest that FBS is not an essential media component, but it may foster differentiation into more mature luminal and myoepithelial cell types.

## Discussion

The utility and relevance of studies of human biology that employ cultured human cells is dependent upon the extent to which the culture systems used accurately reflect the *in vivo* biology and support the multiple cells types present in the native tissue. For HMEC, various media have been reported to support normal growth and differentiated propertied [3, 7–9, 15, 16, 19]. Our laboratory has developed three distinct media: serum-containing MM [15, 16, 19], serum-free MCDB 170 [7, 16], and serum-containing M87A [3, 9]; additional media, such as WIT-P, have been developed by other labs [8]. Prior studies using these media individually suggest that each media will generate significant differences in pre-stasis HMEC. Because the biological impacts of the media on HMEC has not been systematically tested it is difficult to interpret differences in results among investigators. Here we present in parallel comparative studies that are specifically designed to expose media-driven differences. We found that MCDB 170-type and WIT-P, two of the most commonly used and commercially available HMEC media, led to earlier loss of lineage diversity and proliferative potential compared to M87A. Here we describe the impact of three different media, MCDB 170, WIT-P, and M87A, on growth and lineage composition of pre-stasis HMEC cultures derived from the same reduction mammoplasty tissues of two different individuals. M87A maintained lineage diversity for as many as 30 PD. In addition, M87A performed well without serum, maintaining PD and growth rate comparable to the serum-containing M87A, although exhibiting slightly reduced diversity.

MCDB 170 medium is the prototype serum-free HMEC medium upon which MEGM and other commonly used HMEC growth media are based. As previously reported [1], MCDB 170 supported pre-stasis growth for only a few passages, associated with increasing levels of p16 expression. Here we additionally show early loss of lineage diversity in the pre-stasis population. Although most cells stop at stasis in MCDB 170, emergence of rare post-selection cells shown to be p16(-) post-stasis [1, 7], later also referred to as vHMEC [22], can occur. Post-selection cells were considered valuable for HMEC studies because their rapid long-term growth enabled greater potential for experimentation and commercialization. However, subsequent studies showed that post-selection post-stasis HMEC possess significant differences from normal finite HMEC in e.g., gene expression, DNA promoter methylation, and lineage markers; some of these alterations are also seen in tumor-derived cells [3, 12, 21]. Post-selection post-stasis HMEC have been suggested to represent a metaplastic phenotype on the pathway to metaplastic cancer [23–25]. The aberrant post-selection post-stasis HMEC have been commercially available for decades but have been sold and utilized as “normal”, despite the published data describing their deviation from normal and expression of some metaplastic and cancer-associated properties. Aberrant p16(-) post-stasis cultures grown in MCDB 170-type medium have also been the basis for most hTERT-immortalized HMEC lines (e.g., HME1 [27], HMEC/184-hTERT, [28–30], HMLE with SV40T, HMLER with SV40T and H-Ras [31, 32]), consequently also affecting the fidelity with which such lines reflect *in vivo*-like behavior. We hypothesize that the rapid induction of p16 in this media is indicative of stressful culture conditions, which may in turn give rise to the observed ~200 promoter methylation changes, including the p16 locus, that result in the post-selection post-stasis population [22].

WIT-P medium is a serum-free alternative to MCDB 170 that aimed to enable better growth of the luminal lineage [8]. We observed representation of K19+/K14- and CD227+/CD10- LEP cells in WIT-P at 2p that was comparable to luminal populations in M87A, but the luminal cells in WIT-P were lost rapidly, as early as 3p. In our hands, both specimens exhibited a slower increase in p16 expression (a measure of stress) in WIT-P than in MCDB 170 but, as also previously reported [33], cells in WIT-P still stopped at p16(+) stasis many PD prior to cells grown in M87A. In the original description of WIT-P media, the reported growth curve shows a lag in growth and no p16 protein after ~10 PD, which would be consistent with what is observed in MCDB 170-grown cultures undergoing selection [8]. In our hands in MCDB 170, p16 expression first appears in cells when they are temporally close to loss of proliferative potential. We speculate that early loss of the luminal cells and appearance of large vacuolated cells in WIT-P is due to p16-inducing stresses. Our data show that WIT-P media does not avoid early p16-associated stasis nor maintain normal luminal lineages for extended passage.

M87A was developed with the goal of reducing stress in normal HMEC mass cultures in order to extend proliferative potential [3]. We have shown that M87A supports pre-stasis HMEC growth, including myoepithelial, luminal, and progenitor cells for 30–60 PD. We examined pre-stasis HMEC grown in M87A from >36 women and observed inter-individual variation in growth potential and heterogeneity of epithelial lineage markers [5]. Comparisons of transcriptomes, DNA methylomes, and single cell proteomics, have shown that HMEC cultured in M87A are maintained in states that are comparable to uncultured *in vivo* normal HMEC [3, 34, 35]. Here we demonstrate that M87A is the only media of the three tested that maintains LEP for more than 3 passages (up to 8). The presence of the hormone oxytocin in the M87A media may play an important role in supporting more growth and diversity. In the mammary gland *in vivo*, myoepithelial cells express the oxytocin receptor [36], so it is possible that the oxytocin acts through cultured myoepithelial cells to support luminal cell growth. We speculate that growth of HMEC as multi-lineage ensembles provides yet-to-be identified microenvironment components and cell-cell interactions that minimize stress and maintain heterogeneity. Consistent with this hypothesis, LEP survive and proliferate much better on feeder layers of MEP, compared to tissue culture plastic or feeder layers of mammary stromal fibroblasts [34]. Our use of non-dissociated epithelial organoids to initiate all the pre-stasis cultures examined here can additionally provide multi-lineage ensembles. In contrast, the other common method used for primary HMEC culture involves dissociating the organoids prior to plating, which may disrupt microenvironment cues helpful in maintaining lineage diversity. Organoid attachment enables the establishment of mass cultures consisting of luminal, myoepithelial, and progenitor cells, which we speculate is important for the stochastic creation of an ecology that supports multiple epithelial cell types.

In addition to oxytocin, there are numerous other differences between the compositions of the three media compared side-by-side (see S1 File), and it is difficult to know exactly which components lead to the altered properties among pre-stasis HMEC cultures. One distinction is compared to M87A, WIT-P has 200-fold more cholera toxin, which impacts multiple facets of cellular physiology but most notably stimulates cAMP release. The use of FBS in M87A also is a component that is distinct from the two serum-free media.

Withdraw of the FBS component from M87A did not alter the growth rate of HMEC from 3p to 10p, but there was a detectable drop in the amount of differentiated LEP and MEP cells. Possibly, the presence of FBS in HMEC cultures, even the 0.25% that is present in M87A, may impact differentiation more than growth. A recent report of conditions for isolating and maintaining estrogen receptor (ER)-expressing pre-stasis luminal cells in HMEC cultures also used serum to an advantage. That method relied upon establishing HMEC cultures from dissociated epithelial organoids in FAD2 media, which contains 5% FBS, and two different TGF-beta inhibitors. In our hands, ER+ luminal cells could be isolated from cells that migrated from attached organoids in FAD2 media, so likely the altered media rather than dissociation was key (data not shown).

The emergence of p16(-) HMEC in MCDB 170-like and WIT-P media could reflect *in vivo* processes during carcinogenesis, since ~20–30% of breast cancers lack p16 expression [37]. Rare HMEC with silenced p16 also have been shown to exist in vivo in histologically normal breast epithelium [38]. In cancers, loss of p16 expression may be due to multiple types of errors, including mutation and promoter silencing. We have seen that loss of p16 expression in pre-stasis HMEC is common during *in vitro* malignant progression resulting from diverse types of exposures, including the chemical carcinogen benzo(a)pyrene (BaP) [21] as well as growth in the stressful MCDB 170 medium. However, the BaP-post-stasis HMEC do not show the extensive additional promoter methylation seen in the post-selection post-stasis cultures [21]. It is not known whether the spontaneous emergence of p16(-) post-stasis cells in MCDB 170 and WIT-P reflects processes that commonly occur during breast cancer progression.

No experimental system is perfect for every scientific question. With respect to understanding normal human breast biology and breast carcinogenesis, the degree to which model systems accurately reflect the complexity of the *in vivo* biology can impact the extent to which results obtained represent what actually occurs during *in vivo* processes. Cell culture models have an obvious lack of *in vivo*-like microenvironment context, however, the methods and type of media used can also significantly alter the cell’s intrinsic biology, and consequently the relevance of the data obtained for human health. Use of media and cells that are not accurate reflections of normal HMEC *in vivo* has the potential of generating data not representative of actual *in vivo* processes. Using media that can support extensive proliferation of HMEC that maintain the gene expression, epigenetic markers, and lineage phenotypes of the multiple cell types that comprise the *in vivo* tissue is an important starting place for developing accurate model systems. By conducting parallel experiments in the three media, and in addition to prior publications, we demonstrate here that the M87A medium can generate such normal HMEC cultures and is superior in supporting *in vivo*-like properties than the commonly used serum free MCDB 170-like and WIT-P media. For the future, combining optimal media and culture methodologies with new technologies that may support in vivo-like architecture, such as micro-physiological systems, could provide even more accurate human tissue model systems.

## Supporting information

S1 File Formulas for WIT-P, MCDB-170, and M87A. Spreadsheet with ingredients for WIT-P, MCDB-170, and M87A media.
(XLSX)
